# Exploring protocol bias in airway microbiome studies: one versus two PCR steps and 16S rRNA gene region V3 V4 versus V4

**DOI:** 10.1186/s12864-020-07252-z

**Published:** 2021-01-04

**Authors:** Christine Drengenes, Tomas M. L. Eagan, Ingvild Haaland, Harald G. Wiker, Rune Nielsen

**Affiliations:** 1 Department of Thoracic Medicine, Haukeland University Hospital, Bergen, Norway; 2 Department of Clinical Science, Faculty of Medicine, University of Bergen, Bergen, Norway; 3 Department of Microbiology, Haukeland University Hospital, Bergen, Norway

**Keywords:** Microbiota, Contamination, Low biomass, Respiratory, 16S rRNA gene

## Abstract

**Background:**

Studies on the airway microbiome have been performed using a wide range of laboratory protocols for high-throughput sequencing of the bacterial 16S ribosomal RNA (16S rRNA) gene. We sought to determine the impact of number of polymerase chain reaction (PCR) steps (1- or 2- steps) and choice of target marker gene region (V3 V4 and V4) on the presentation of the upper and lower airway microbiome. Our analyses included lllumina MiSeq sequencing following three setups: Setup 1 (2-step PCR; V3 V4 region), Setup 2 (2-step PCR; V4 region), Setup 3 (1-step PCR; V4 region). Samples included oral wash, protected specimen brushes and protected bronchoalveolar lavage (healthy and obstructive lung disease), and negative controls.

**Results:**

The number of sequences and amplicon sequence variants (ASV) decreased in order setup1 > setup2 > setup3. This trend appeared to be associated with an increased taxonomic resolution when sequencing the V3 V4 region (setup 1) and an increased number of small ASVs in setups 1 and 2. The latter was considered a result of contamination in the two-step PCR protocols as well as sequencing across multiple runs (setup 1). Although genera *Streptococcus*, *Prevotella*, *Veillonella* and *Rothia* dominated, differences in relative abundance were observed across all setups. Analyses of beta-diversity revealed that while oral wash samples (high biomass) clustered together regardless of number of PCR steps, samples from the lungs (low biomass) separated. The removal of contaminants identified using the Decontam package in R, did not resolve differences in results between sequencing setups.

**Conclusions:**

Differences in number of PCR steps will have an impact of final bacterial community descriptions, and more so for samples of low bacterial load. Our findings could not be explained by differences in contamination levels alone, and more research is needed to understand how variations in PCR-setups and reagents may be contributing to the observed protocol bias.

**Supplementary Information:**

The online version contains supplementary material available at 10.1186/s12864-020-07252-z.

## Background

The bacterial airway microbiome has been studied using a wide range of protocols for high-throughput sequencing of the bacterial 16S ribosomal RNA (16S rRNA) gene. Common to all amplicon based protocols is the application of the polymerase chain reaction (PCR) for i) amplification of the target marker gene to be sequenced and ii) the addition of index sequences necessary for sample multiplexing. These steps can be performed in a single PCR or in two separate PCRs. No study has addressed whether the increased number of laboratory processing steps associated with a 2-step PCR protocol, will leave samples more vulnerable to bacterial DNA contamination from the laboratory than when following a 1-step PCR protocol. The inverse relationship between sample bacterial load and the impact of contamination has been well documented in the literature by others [1, 2] and ourselves [3]. Thus, we predicted that while samples with a high bacterial load (i.e. upper airway samples) would be able to buffer against protocol effects resulting from differences in contamination levels, samples with a low bacterial load (i.e. lower airway samples) would not be resistant to these effects.

In addition to number of PCR steps, sequencing protocols vary by choice of targeted marker gene region. Several different 16S rRNA gene variable regions have been targeted in studies of the lung microbiome, including V1 V2 [4, 5], V1 V3 [6–8], V3 V5 [7, 9–13], V3 [14, 15] and V4 [16–19]. Choice of target marker gene region has been limited by the short length of DNA that can be sequenced using the most common high-throughput sequencing technologies. The V4 region has increased in popularity as studies on estimates of alpha- [20] and beta- diversity [21] (i.e. measures of diversity within and between samples, respectively) and taxonomic assignments [22, 23] have collectively indicated that this site generates the most accurate descriptions. In addition, its relatively short length has allowed for the complete overlap of the forward and reverse sequencing read; advantageous because correction of sequencing errors is possible using the read with highest quality score [24]. The increased capacity of the MiSeq sequencer to sequence longer DNA sequences coupled with the development of novel denoising strategies (e.g. DADA2 [25]), has however led to an increased interest in the targeting of the longer V3 V4 region. It is however unclear how these results compare to earlier studies based on the shorter V4 region.

In the current study, we sought to i) evaluate the impact of bacterial DNA contamination when processing samples through protocols that vary in number of PCR steps (1- or 2-steps) and ii) determine the impact of choice of target marker gene region (V3 V4 vs V4) on the presentation of the upper and lower airway microbiome. To address these issues we processed samples of both high and low bacterial load through three library preparation setups varying in the number of PCR steps and target marker gene region: Setup 1 (2-step PCR; V3 V4 region), Setup 2 (2-step PCR; V4 region), Setup 3 (1-step PCR; V4 region). The upper airways were represented by oral wash (OW) samples and the lower airways by protected specimen brushes (PSB) and protected bronchoalveolar lavages (PBAL) collected by bronchoscopy. Negative control samples (NCS) consisting of saline used in the collection of all samples was processed together with the clinical samples for assessment of contamination.

## Results

### Study participants

The study included 23 subjects from the MicroCOPD study [26]. Subject characteristics are provided in Table 1.

**Table 1:** Subject characteristics

	Controls	COPD	Asthma
**Subjects**	9	10	4
**Age, mean** ± SD years	63.0 ± 6.7	68.2 ± 5.2	63.6 ± 3.1
**Men**	6 (66.7%)	8 (80.0%)	2 (50.0%)
**Current-smokers**	2 (22.2%)	1 (10.0%)	0
**Former-smokers**	5 (55.6%)	9 (90.0%)	3 (75.0%)
**Never-smokers**	2 (22.2%)	0	1 (25.0%)
**Smoker pack years, mean** ± SD years	11.8 ± 6.1	25.2 ± 8.1	12.1 ± 6.2
**FEV**_**1**_ **(% predicted), mean** ± SD	97.0 ± 13.7	72.6 ± 23.2	101.6 ± 9.3
**Inhaled corticosteroids**	0	2 (20.0%)	3 (75.0%)
**LABA**	0	3 (30.0%)	1 (25.0%)
**LAMA**	0	4 (40.0%)	0

*COPD* Chronic obstructive pulmonary disease; *FEV*_*1*_ Forced expiratory volume in 1 s; *LABA* Long-acting beta-agonist; *LAMA* Long-acting muscarinic antagonist. 1 smoker pack year = 20 cigarettes (one pack) smoked daily for 1 year. Age, smoker pack years and FEV_1_ (% predicted) are presented as the mean ± standard deviation.SD: standard deviation.

### Number of sequences and amplicon sequence variants (ASVs)

**Fig. 1:**
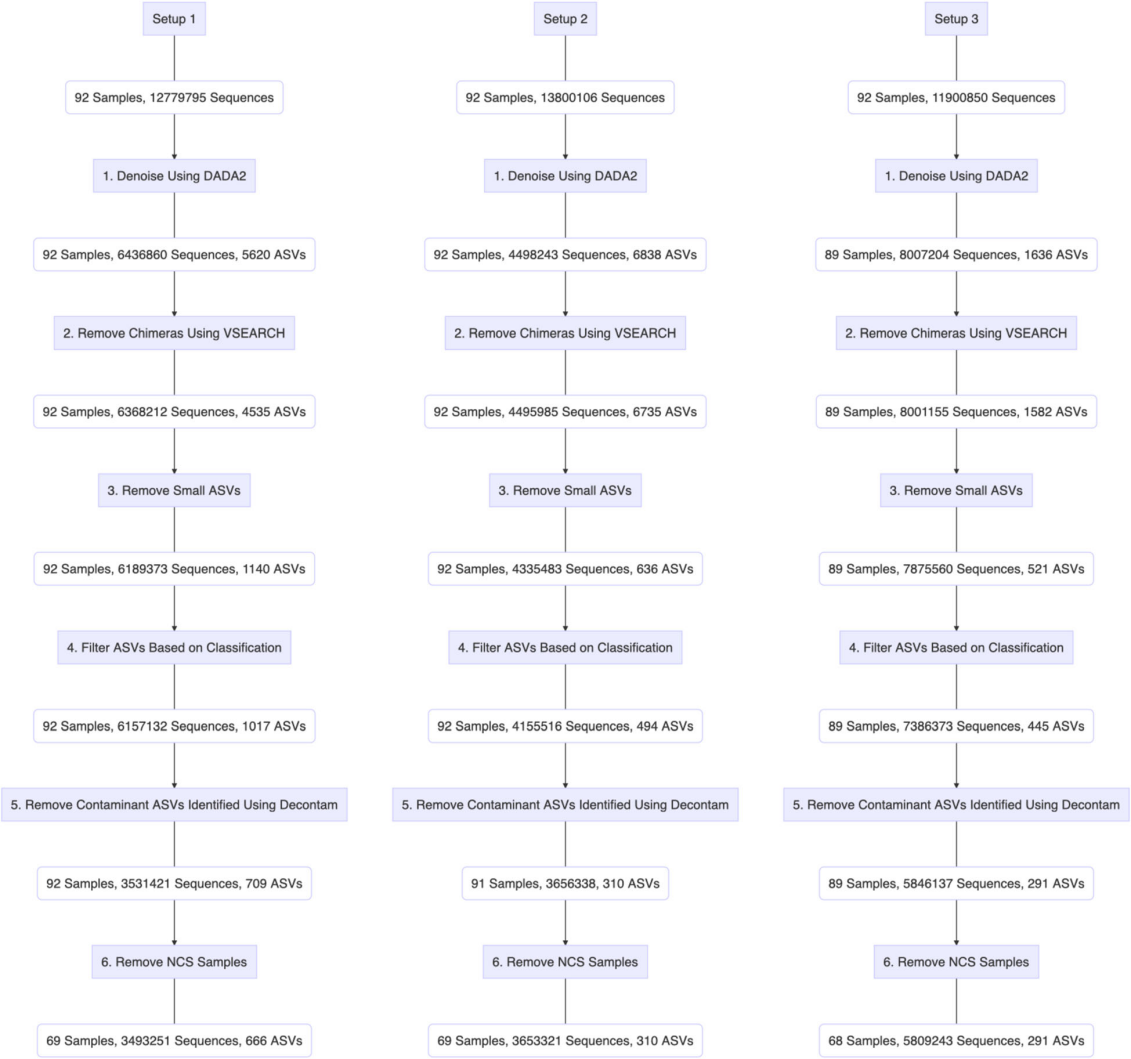
Comparison of the number of sequences and amplicon sequence variants (ASVs), retained at each bioinformatic filtering step for procedural samples (PSB, PBAL, OW, NCS) collected from 23 participants (*n* = 92 samples). Setup 1 (2-step PCR; V3 V4 region), Setup 2 (2-step PCR; V4 region), Setup 3 (1-step PCR; V4 region)

We began our analyses with a comparison of the number of sequences and amplicon sequence variants (ASVs) retained at each step when processing through the bioinformatic pipeline (Fig. 1). For sequencing setup 1, the procedural samples were dispersed across four sequencing runs (I-IV). For sequencing setups 2 and 3, two separate sequencing runs (one per setup) were conducted including all samples.

As the sequences were passed through the different bioinformatic filtering steps, the total number of sequences and ASVs across the three setups became more similar. Denoising in DADA2 (Fig. 1, step 1) resulted in the greatest decrease in sequence number. The greatest decrease in ASV number occurred after the removal of *small* ASVs, for which the number of sequences was calculated to be less than 0.005% of the total number of sequences on the same run (Fig. 1, step 3). The drop in ASV number was greatest for sequencing setups 1 and 2, both of which are based on the longer 2-step PCR protocol.

After the final filtering step (Fig. 1, step 6), the number of ASVs was significantly higher for setup 1 compared to that observed for setups 2 and 3. When we restricted analyses to samples from the largest sequencing run in setup 1 (14 participants, 56 samples) (Additional File 1: Fig. S.1.), the number of ASVs for setup 1 was now more comparable to that observed for setups 2 and 3 (Additional File 1: Fig. S.1., step 6). The higher number of ASVs still observed for setup 1, was expected due to the greater taxonomic resolution obtained when targeting a longer marker gene region (V3 V4).

### Protocol effects on mock community sample

The mock community sample HM-783D, consisting of genomic DNA from 20 different bacterial species (17 genera) was included on each sequencing run. For a detailed presentation of the mock community, see Additional File 7: Supplementary Methods. Because the protocols targeting different hypervariable regions result in different ASVs, we describe ASVs obtained for setup 1 (V3 V4 target) and setups 2 and 3 (V4 target), separately.

When following setup 1 across four sequencing runs, we obtained the following number of sequences and ASVs: run I: 128,413 (27 ASVs); run II: 109,709 (23 ASVs); run III: 110,492 (24 ASVs) and run IV: 84,909 (27 ASVs). As the number of sequences obtained for each run was similar, ASV numbers were also comparable across the four runs. While most genera were defined by a single ASV, genera *Escherichia*, *Staphylococcus*, *Streptococcus*, *Clostridium* and *Rhodobacter* were defined by multiple ASVs. The major ASVs attributed to each genus (i.e. those with the highest number of sequences) were the same across all four sequencing runs. For a detailed presentation of the ASVs observed in the mock community following setup 1, see Additional File 2: Table S.1.

When following setups 2 and 3, we obtained 103,409 sequences (31 ASVs) and 120,073 sequences (23 ASVs), respectively. The genera *Escherichia*, *Staphylococcus*, *Streptococcus*, *Clostridium* and *Neisseria* were defined by multiple ASVs. The major ASVs attributed to each genus were the same in both setups 2 and 3. For a detailed presentation of the ASVs observed in the mock community following each setup, see Additional File 3: Table S.2. and Additional File 4:Table S.3.

**Fig. 2:**
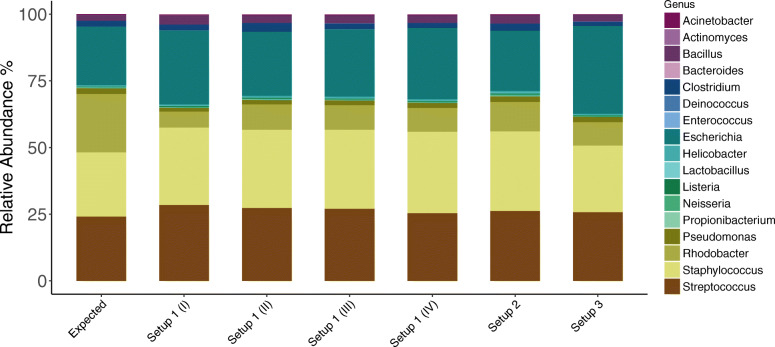
Analysis of mock community HM-783D. The expected relative abundances of genera in the mock community sample is presented next to that observed in the sequencing output across the three setups. The *Escherichia* genus consisted of ASVs classified to family level (*Enterobacteriaceae*); ASV ffc36e27c82042664a16bcd4d380b286 dominated Setup 1 targeting the 16S rRNA gene V3 V4 region and ASV d46e2205f0c6ecf67b51f83d111c509c dominated Setups 2 and 3 targeting the V4 region. Using the NCBI blastn tool we were able to confirm that these ASVs belonged to the *Escherichia coli* genus. Bioinformatics processing steps were limited to DADA2, VSEARCH, taxonomy assignment and removal of features not classified at minimum to phylum level. Setup 1 (2-step PCR; V3 V4 region), Setup 2 (2-step PCR; V4 region), Setup 3 (1-step PCR; V4 region)

A summary of the expected and observed taxonomic distribution in the mock community sample, obtained for each setup is presented in Fig. 2 and Table 2. We found that the three sequencing setups were for the most part equally efficient at recovering high abundant mock community members. Sequencing setup 3, was least efficient at recovering the low abundant members. Across all setups, we observed an increase in the relative abundances of genera *Escherichia* and *Staphylococcus* and a significant decrease in *Rhodobacter* compared to that expected. All setups generated low abundant ASVs that did not match to any of the expected taxa in the mock community (i.e contaminants). Because the mock community sample was included on each of the four sequencing runs I-IV performed following setup 1, we were also able to show that mock community sequencing is reproducible.

**Table 2:** Expected and observed relative abundance (%) of genera in mock community sample HM-783D. Setup 1 (2-step PCR; V3 V4 region); Setup 2 (2-step PCR; V4 region); Setup 3 (1-step PCR; V4 region)

Genera	Expected	Setup 1 (I)	Setup 1 (II)	Setup 1 (III)	Setup 1 (IV)	Setup 2	Setup 3
*Escherichia*	21.91	27.68	23.99	25.20	26.65	22.54	32.90
*Rhodobacter*	21.91	5.98	9.52	9.23	8.94	11.00	8.77
*Staphylococcus*	24.10	29.02	29.27	29.66	30.56	29.88	24.98
*Streptococcus*	24.12	28.51	27.39	27.03	25.38	26.20	25.81
*Bacillus*	2.19	3.38	2.86	2.95	2.85	3.15	2.49
*Clostridium*	2.19	2.18	3.28	2.19	1.88	2.64	1.69
*Pseudomonas*	2.19	1.44	1.68	1.75	1.93	2.12	2.02
*Acinetobacter*	0.22	0.32	0.29	0.33	0.30	0.29	0.12
*Helicobacter*	0.22	0.36	0.49	0.44	0.38	0.61	0.26
*Lactobacillus*	0.22	0.22	0.20	0.23	0.24	0.35	0.18
*Listeria*	0.22	0.33	0.33	0.30	0.32	0.37	0.26
*Neisseria*	0.22	0.24	0.31	0.30	0.27	0.43	0.39
*Propionibacterium*	0.22	0.13	0.22	0.18	0.15	0.29	0.00
*Actinomyces*	0.02	0.01	0.01	0.00	0.00	0.01	0.00
*Bacteroides*	0.02	0.02	0.00	0.03	0.02	0.04	0.02
*Deinococcus*	0.02	0.02	0.04	0.03	0.02	0.03	0.02
*Enterococcus*	0.02	0.03	0.02	0.03	0.02	0.02	0.00
Other	0.00	0.13	0.11	0.10	0.09	0.03	0.08

### Protocol effects on contamination profiles

Our working hypothesis linked protocol bias to differences in susceptibility to laboratory contamination. We therefore proceeded with an examination of the average top 20 ASVs found in NCS. Because the same DNA extracts were processed through each of the three setups, any observed differences in taxonomic distribution would be attributed to library preparation steps (post DNA extraction). We also examined PCR water samples included on each sequencing run. In contrast to NCS, this later sample reflects contamination introduced during library preparation steps without interference from contaminating DNA introduced from the DNA extraction kit. ASVs obtained for setups 2 and 3, targeting the V4 region and the single setup targeting the V3 V4 region are described separately.

**Fig. 3:**
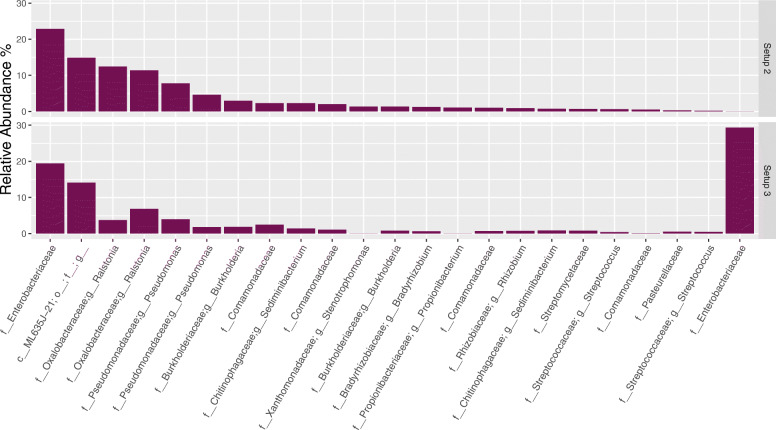
Comparison of the 20 most abundant amplicon sequence variants (ASVs) observed in negative control samples (NCS) after sequencing following setups 2 and 3. Taxa presented according to decreasing abundance for ASVs observed following setup 2. Bioinformatic processing steps were performed up the removal of contaminants identified using Decontam. Taxonomic rank is described using prefixes (*c__*: class, *o__*: order, *f__*: family, *g__*: genus). Setup 2 (2-step PCR; V4 region); Setup 3 (1-step PCR; V4 region). Data is presented as the average relative abundance. Data unrarefied

The average top 20 ASVs observed in NCS in setups 2 and 3, are presented in Fig. 3. The samples were dominated by many of the same taxa, and most of these taxa were defined by the same ASVs. The Decontam package (method = either, threshold = 0.5) applied downstream of the presented data identified the majority of the top 20 ASVs presented in NCS as contaminants. Exceptions included both ASVs mapping to the genus *Streptococcus* (in line with our previous findings [3]) (using NCBI blastn these ASVs were determined to be *Streptococcus oralis* (06f825b512d903b9230e1a55d87359ee) and *Streptococcus thermophilus* (fd496fd32dc8c08ade2e8b6c9d8ee13d) and the single ASV mapping to the family *Pasteurellaceae*.

The distribution of ASVs in NCS (Fig. 3) differed the most between setups 2 and 3 for an ASV belonging to the family *Enterobacteriaceae* (mapped to *Escherichia* using NCBI blastn), with a significant increase observed in samples sequenced by setup 3 (0.02% observed for setup 2 and 29.34% observed for setup 3). These findings were in accordance with the results from the mock community analysis (Fig. 2), for which the same *Escherichia* ASV was also found at higher levels in the mock community sample sequenced by setup 3 (22.54% observed for setup 2 and 32.90% observed for setup 3). Its relatively high abundance in the mock community processed through setup 2 compared to NCS was expected as the *Escherichia* genus defined by this ASV constituted 21.91% of the expected mock community profile; i.e. for this sample the ASV represented both a contaminant and a non-contaminant.

We proceeded with a comparison of the taxonomic distribution in PCR water samples sequenced following setups 2 and 3 (Table 3). A relatively low number of sequences and ASVs were obtained (setup 2: 178 sequences (10 ASVs); setup 3: 130 sequences (6 ASVs)). Importantly, the dominating ASV (35.38%) found in the PCR water samples sequenced following setup 3, was the same ASV mapping to *Escherichia* discussed above. The same ASV was not found in the PCR water sample sequenced by setup 2. Together these findings indicate that the *Escherichia* ASV is a contaminant introduced during steps of library preparation using a reagent that is exclusive to setup 3.

**Table 3:** Relative abundance (%) of ASVs observed in PCR water samples in setups 2 and 3

ASV	Lowest Classification	Setup 2	Setup 3
06f825b512d903b9230e1a55d87359ee	f__Streptococcaceae; g__Streptococcus	35.39	20.77
ddfd49f939f92958b1ec816741055348	f__Oxalobacteraceae; g__Ralstonia; s__	12.36	0.00
394eda29c886632f514dd94b58381186	f__Pasteurellaceae	8.99	0.00
d32e579b3ae7b2aae8d5bf9f027c29af	f__Comamonadaceae	8.99	0.00
5648dccee530d68ceb3e4d7d22cf8756	f__Pseudomonadaceae; g__Pseudomonas	7.87	0.00
4f5efd25dacb5d639316e7291ff6ff8b	f__Neisseriaceae; g__Neisseria	7.87	7.69
85c44c83eddc5d3028261a1000b7d0e1	f__Gemellaceae	5.62	0.00
923f521b9cf313f1f95c9367e09bbc1c	f__Veillonellaceae; g__Veillonella; s__dispar	5.62	12.31
dcba105f35d8ebc9e22269c7491ad3a7	f__Xanthomonadaceae; g__Stenotrophomonas; s__geniculata	5.06	0.00
df8456a1abbfb4c8a2c450b44378d4cb	f__Actinomycetaceae; g__Actinomyces; s__	2.25	0.00
d46e2205f0c6ecf67b51f83d111c509c*	f__Enterobacteriaceae	0.00	35.38
edc9e5c16e40aff1eadce6597940f08f	f__Streptococcaceae; g__Streptococcus; s__	0.00	13.85
65d43491988bfe557da4d86a5ba25dae	f__Staphylococcaceae; g__Staphylococcus	0.00	10.00

*Escherichia ASV also observed to differentiate mock community samples and NCS in setups 2 and 3. Bioinformatic processing steps were performed up until the removal of contaminants identified using Decontam. Taxonomic rank is described using prefixes (f__: family, g__: genus, s__: species)

**Fig. 4:**
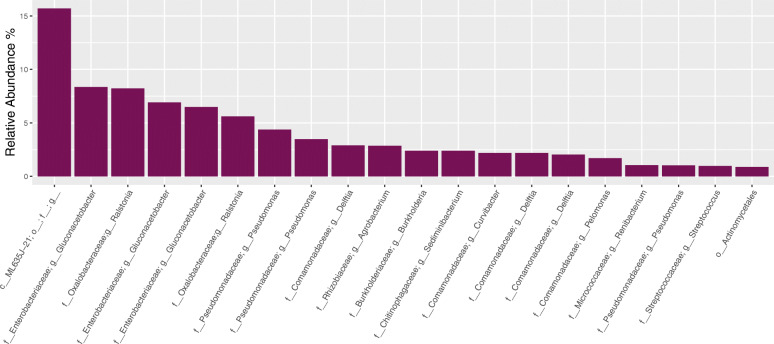
The 20 most abundant amplicon sequence variants (ASVs) observed in negative control samples (NCS) after sequencing following setup 1. Multiple ASVs mapped to genera *Gluconacetobacter,* belonging to familiy *Enterobacteriaceae* (cummulative 22%). Bioinformatic processing steps were performed up until the removal of contaminants identified using Decontam. Taxonomic rank is described using prefixes (*c__*: class, *o__*: order, *f__*: family, *g__*: genus). Data is presented as the average relative abundance. Data unrarefied

We next looked at the average top 20 ASVs observed in NCS when sequencing following setup 1 (Fig. 4). The taxonomic profiles obtained after sequencing the longer V3 V4 region resulted in greater taxonomic resolution compared to that observed when sequencing the V4 region in setups 2 and 3. Whereas the three ASVs belonging to the family *Enterobacteriaceae* classified down to genus level *Gluconacetobacter* in setup 1, the *Enterobacteriaceae* ASVs classified no lower than to family level in setups 2 and 3 (Fig. 3). The cumulative average relative abundance of the three ASVs mapping to *Gluconacetobacter* when following setup 1 (22%) was however the same as that found for the single ASV mapping to the family *Enterobacteriaceae* when following setup 2 (23%). Thus, for these two setups, the contamination profiles were similar although greater resolution was obtained when sequencing a longer target gene region in setup 1 (V3 V4).

### Protocol effects on procedural samples

We next compared the sequencing output obtained for the procedural samples sequenced following each of the three setups. Because we suspected that any differences observed between sequencing setups could be explained by differences in susceptibility to laboratory contamination, comparisons were made both before and after the removal of contaminants identified in Decontam (Fig. 1, Step 5).

**Fig. 5:**
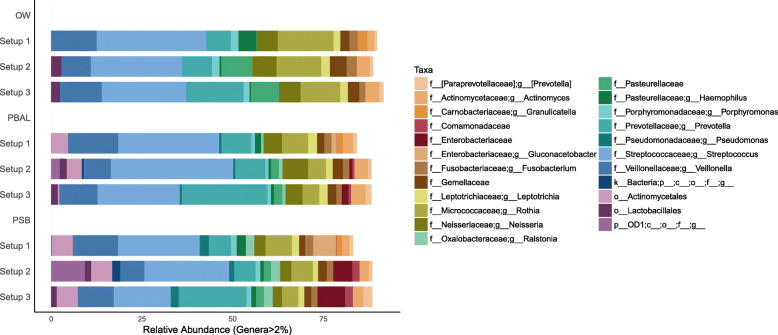
Taxonomic distribution obtained for procedural samples before the removal of Decontam contaminants. ASVs attributed to the family *Enterobacteriaceae*, had dominated the NCS across all setups. In the procedural samples, *Enterobacteriaceae* was observed with the following relative abundances in setups 2 and 3: setup 2 (OW: 0%; PBAL: 0.83%; PSB: 5.23%); setup 3 (OW: 0.01%; PBAL: 1.87%; PSB: 7.51%). ASVs attributed to the genus *Gluconacetobacter* within the family *Enterobacteriaceae* was observed in procedural samples with the following relative abundances in setup 1 (OW: 0%; PBAL: 1.42%; PSB: 6.32%). Samples with fewer than 1000 sequences had been omitted from the analyses leaving the following number of samples in each setup: Setup 1 (OW: *n* = 22; PBAL: *n* = 23; PSB: *n* = 23); Setup 2 (OW: *n* = 23; PBAL: *n* = 23; PSB: *n* = 23); Setup 3 (OW: *n* = 23; PBAL: *n* = 21; PSB: *n* = 22). Setup 1 (2-step PCR; V3 V4 region); Setup 2 (2-step PCR; V4 region); Setup 3 (1-step PCR; V4 region). Taxonomic rank is described using prefixes (p__: phyla; c__: class; o__: order; f__: family; g__: genus)

Before the removal of Decontam contaminants (Fig. 5), we found that across all three sequencing setups, procedural samples (OW, PSB, PBAL) were dominated by many of the same taxa. The most prominent taxa averaged across all samples in order of decreasing relative abundance were genera *Streptococcus*, *Prevotella*, *Veillonella* and *Rothia*. We interpreted these as representative of the authentic airway microbiota based on the growing body of literature for which these same taxa have been consistently observed in airways.

Several less abundant taxa for which we interpreted as contaminants, based on their dominance in NCS were also observed in the data. We previously learned that ASVs attributed to the family *Enterobacteriaceae* dominated the NCS and that an ASV mapping to *Escherichia* had a discriminating impact on NCS and mock communities processed through setup 3. We were therefore particularly interested in understanding whether *Enterobacteriaceae* would also have a discriminating impact on procedural samples processed through the different sequencing setups. Across all three sequencing setups we found that the levels of *Enterobacteriaceae* was highest in samples from the lower airways (PSB > PBAL) and nearly undetected in OW samples (Fig. 5). The higher levels of *Enterobacteriaceae* in PSB samples compared to PBAL, was expected as less sample volume was used as input to the DNA extraction protocol (450 μl PSB vs 1800 μl PBAL) thereby securing a lower bacterial load in PSB compared to PBAL. Across all sample types, the relative abundance of *Enterobacteriaceae* was highest when sequencing following setup 3; this was also in accordance with our results when sequencing the mock community and likely due to the additional *Escherichia* contamination introduced during library preparation following setup 3 (Fig. 3). The relative abundance of *Enterobacteriaceae* per sample is provided in the Additional File 5: Table S.4. By analysis of beta-diversity using the *unweighted* UniFrac metric, we were able to confirm that there was greater overlap or similarity between the bacterial communities found in NCS and procedural samples from the lungs when sequencing following setup 3 (Additional File 6: Fig. S.2).

**Fig. 6:**
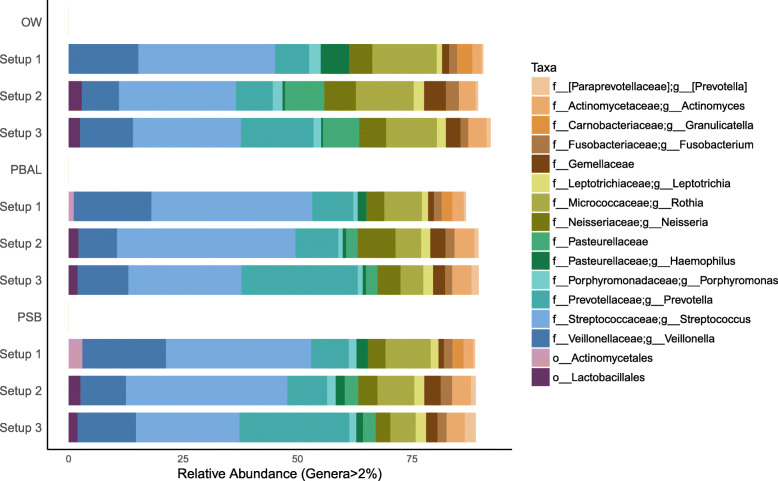
Taxonomic distribution obtained for procedural samples after the removal of Decontam contaminants. Samples with fewer than 1000 sequences were omitted. Number of samples in each setup: V3V4 protocol A (OW: *n* = 22; PBAL: *n* = 22; PSB: *n* = 21), V4 protocol A (OW: *n* = 23; PBAL: *n* = 22; PSB: *n* = 20); V4 protocol B (OW: *n* = 23; PBAL: *n* = 21; PSB: *n* = 21). Setup 1 (2-step PCR; V3 V4 region); Setup 2 (2-step PCR; V4 region); Setup 3 (1-step PCR; V4 region). Taxonomic rank is described using prefixes (p__: phyla; c__: class; o__: order; f__: family; g__: genus)

After the removal of Decontam contaminants, the less abundant taxa that we predicted as representative of contaminants had been filtered out (Fig. 6). Although the dominating taxa across all samples were now mainly expected core airway microbiota members, the relative abundances of these taxa still varied across the three setups.

**Fig. 7:**
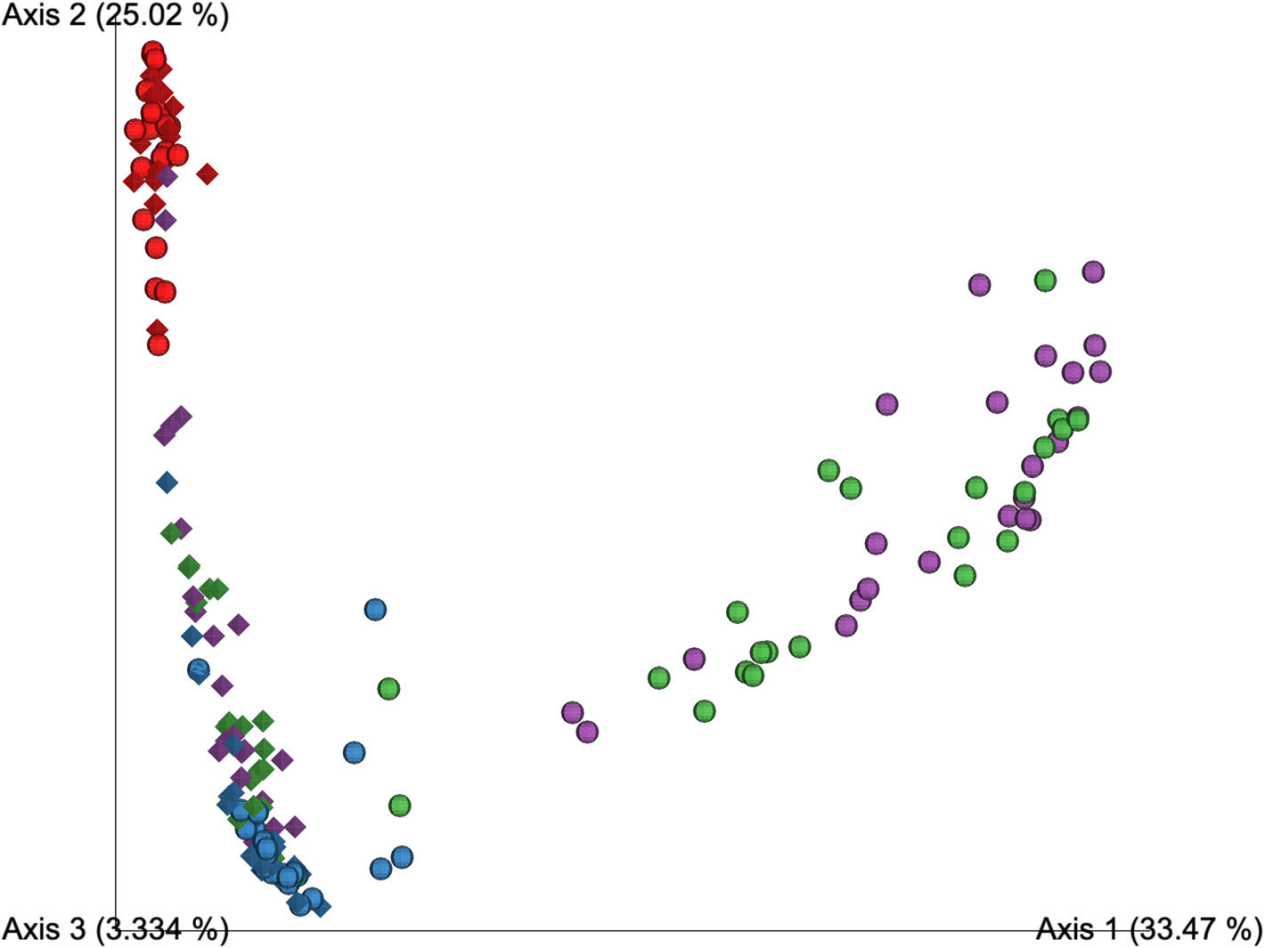
Principal coordinates analysis on *unweighted* UniFrac distances for procedural samples sequenced following setup 2 (sphere) and 3 (diamond) *before* the removal of Decontam contaminants. Rarefaction depth: 1066 sequences. Setup 2 samples include OW: *n* = 23; PBAL: *n* = 23; PSB: *n* = 23; NCS: *n* = 21 and setup 3 samples include OW: *n* = 23; PBAL: *n* = 21; PSB: *n* = 22; NCS: *n* = 18. Oral Wash (OW): blue; Protected bronchoalveolar lavage (PBAL): green; Protected specimen brushes (PSB): purple; Negative control samples (NCS): red. Setup 2 (2-step PCR; V4 region), Setup 3 (1-step PCR; V4 region)

**Fig. 8:**
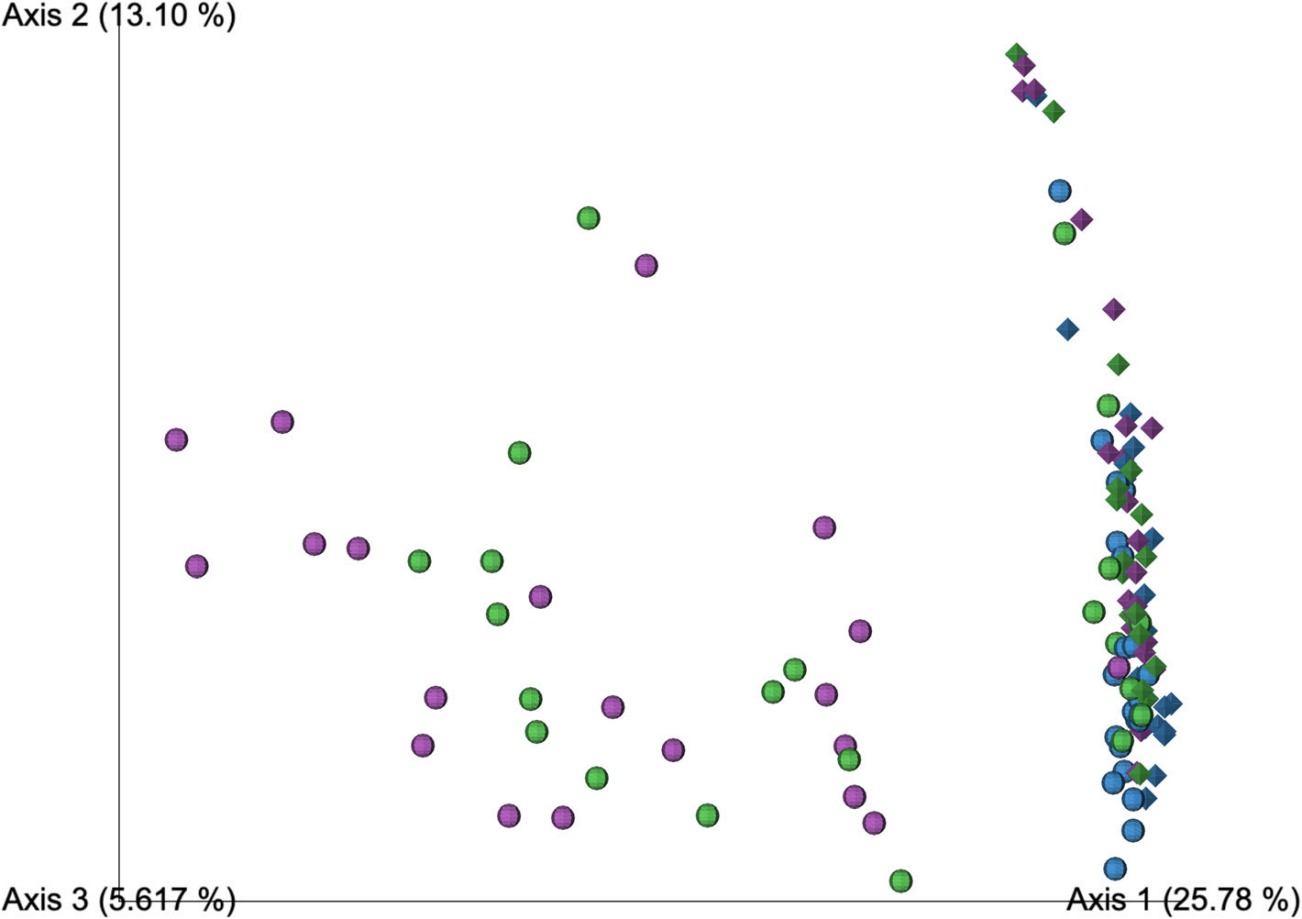
Principal coordinates analysis on *unweighted* UniFrac distances for procedural samples sequenced following setup 2 (sphere) and 3 (diamond) *after* the removal of Decontam contaminants. Rarefaction depth: 1139 sequences. Setup 2 samples include OW: *n* = 23; PBAL: *n* = 22; PSB: *n* = 20 and setup 3 samples include OW: *n* = 23; PBAL: *n* = 21; PSB: *n* = 21. Oral Wash (OW): blue; Protected bronchoalveolar lavage (PBAL): green; Protected specimen brushes (PSB): purple. Setup 2 (2-step PCR; V4 region), Setup 3 (1-step PCR; V4 region)

A direct comparison of the bacterial communities recovered when sequencing by a 1 or 2 step PCR protocol was achieved by analysis of beta-diversity (using the *unweighted* UniFrac metric) on samples processed through each of setups 2 and 3. Before the removal of Decontam contaminants, OW and NCS clustered together regardless of whether they had been processed through setups 2 or 3 (Fig. 7). The samples from the lungs however clustered separately according to the protocol for which they were processed. When Decontam contaminants were removed, the samples from the lungs processed by setups 2 and 3 became more similar in bacterial community composition, as indicated by a greater degree of overlap in PCoA space (Fig. 8). The separation of the lower airway samples based on the setup for which they were processed was however still apparent. This indicated that mechanisms related to the low bacterial load, other than differences in contamination were driving the observed protocol bias. For analysis of beta-diversity using the *weighted* UniFrac metric before and after removal of Decontam contaminants, see Additional File 8: Fig. S.3 and Additional File 9: Fig. S.4, respectively.

## Discussion

We have shown that choice of library preparation protocol for high-throughput amplicon-based sequencing of the 16S rRNA gene (1-step PCR vs 2-step PCR) will have an impact on final bacterial community descriptions for airway samples - and more so for samples of low bacterial load. Differences observed when sequencing the different target regions (V3 V4 and V4) appeared to be relatively small in comparison, and mainly attributed to differences in taxonomic resolution. Using bioinformatic filtering parameters, we were able to reduce but not completely remove the differences in sequencing output observed for the three sequencing setups: Setup 1 (2-step PCR; V3 V4), Setup 2 (2-step PCR; V4) and Setup 3 (1-step PCR; V4). We propose that protocol bias in studies of the lung microbiome are related not only to differences in susceptibility to contamination but also to less understood (and largely ignored) mechanisms of PCR bias.

Beginning with a comparison of the number of sequences and ASVs retained at each bioinformatic processing step, we gained insight into the differences in the sequencing output generated for each of the three setups. We found that the removal of small ASVs resulted in the greatest decrease in total ASV number across all three setups - with greatest impact on data generated from the two sequencing setups based on the 2-step PCR protocol (setup 1 and 2). Our interpretation was that the small ASVs likely represent low abundant contamination and that the observed higher frequencies in data generated when processing through longer laboratory workflows was as predicted. Interestingly, this filtering step was originally recommended for filtering out spurious operational taxonomic units (OTUs) derived from PCR and sequencing error [27], and therefore not regarded as necessary after denoisning to ASVs [28]. The total number of ASVs after the removal of small ASVs, was still markedly higher when sequencing was performed following setup 1, for which samples were spread across four different sequencing runs. We can expect contamination profiles to vary across sequencing runs, thereby adding to the number of ASVs in the data set, and we therefore interpreted the higher number of ASVs as contamination that had not been filtered out. When analyses were conducted on the subset of samples sequenced on the same run, we still observed a slight increase in ASV count in setup 1; this likely attributed to the greater taxonomic resolution obtained when sequencing a larger gene region. Based on the raw sequencing data, the take home message is therefore that researchers need to pay particular attention to small ASVs when making comparisons across datasets sequenced following different protocols. The observed inflation of ASVs when sequencing across multiple sequencing runs also needs to be accounted for.

By sequencing of a mock community sample, we were able to show that the three sequencing setups were for the most part equally efficient at recovering the high abundant mock community members. For reasons that are unclear to us, we found that sequencing setup 3, was least efficient at recovering the low abundant members. Together with the observation that the total number of ASVs recovered following setup 3 was lower than for Setups 1 and 2, we concluded that the 1 step-PCR protocol may be less apt for detecting rare but potentially significant taxa [29, 30]. Berry et al. [31] also compared sequencing data generated when processing samples through PCR protocols that differed in the number of PCR steps (1-step PCR vs 2-step PCR). In accordance with our findings, they observed reduced richness when processing samples through the 1-step PCR protocol. Thus, it could be that although the 1-step PCR protocol may generate data less influenced by small contaminating ASVs, measures of alpha diversity may be underestimated.

To further explore the potential impact of contamination, we compared the contamination profiles (based on NCS) obtained for the three sequencing setups. We were surprised to find that the NCS samples processed through setup 3 were dominated by an ASV mapping to *Escherichia coli* (family *Enterobacteriaceae*). It was unexpected because we have previously traced the main source of contamination in the MicroCOPD study to the DNA extraction kit [3]. Because the same DNA extracts were used as input into the sequencing setup 3, we expected that the lower number of laboratory processing steps compared to setups 1 and 2, would secure a contaminant profile representative of that introduced during DNA extraction. We however learned that a contaminant introduced during library preparation was enough to overwhelm the contamination profile of the entire sequencing run. We immediately suspected that the DNA polymerase, manufactured in *Escherichia coli* and used exclusively in the PCR amplification step when sequencing following setup 3, was the main contamination source. Our findings emphasize the fact that researchers must be meticulous in their choice of PCR reagents and also aware of these effects when comparing data generated using different protocols.

We have previously determined the sample bacterial load for the samples included in the current study, and estimated that contaminants will represent 10–50% of the sequencing output for lower airway samples when sequencing by setup 1 [3]. We found that the *Enterobacteriaceae* family represented less than 10% of the taxonomy profiles for the procedural samples in all three setups and recognized that a significant fraction of the contaminants, were likely also represented by small ASVs and other taxa. For a more accurate assessment of the impact of contamination, we therefore also relied on the Decontam R package [32] for the identification of contaminants. We predicted that if contamination was the main distinguishing factor causing the separation in sequencing output across sequencing setups, the removal of Decontam contaminants would close this gap. By analysis of *unweighted* Unifrac distances in PCoA space, both before and after the removal of Decontam contaminants, we observed that while the high biomass OW samples clustered together, the low biomass samples from the lungs (PBAL,PSB) separated according to the setup 2 or 3, for which they had been processed. We concluded that factors related to bacterial load, other than contamination must also be contributing to the observed protocol bias.

The polymerase chain reaction (PCR) lies at the core of all amplicon-based sequencing protocols. The impact of PCR related bias (i.e. all mechanisms that may lead to the preferential amplification of particular sequences or taxa) on studies involving samples holding a low bacterial load is however not well understood. This despite that recent papers as well as research dating back even two decades has documented that PCR related bias appears to increase with decreasing template DNA concentration [1, 33–35]. Kennedy et al. [35] observed that bacterial community profiles of replicate soil samples decreased in similarity after sample dilution. The authors attributed these observations to an increased impact of stochastic fluctuations in PCR amplifications at lower bacterial loads. Biesbroek et al. [1] observed an increase in *Firmicutes* and decrease in *Bacteriodetes* across a serially diluted saliva sample, but were unable to explain the direct mechanism behind their observations. Our study contributes to the literature addressing these issues by demonstrating that samples of high bacterial load (OW) appear to be able to buffer against protocol bias (i.e. differences in number of PCR steps), while samples of low bacterial load (PSB, PBAL) are directly impacted. More research is needed in order to understand the extent to which these mechanisms are responsible for our observations.

The results presented in the current study have several important implications. Because the upper respiratory tract represents both i) a major potential source of contamination under sampling and ii) the main source community for the lung microbiota, most studies include representative samples from this site (e.g. OW samples) [4, 17, 19, 36, 37]. Our findings demonstrate that the observed overlap between the bacterial communities of the upper and lower respiratory tract may be protocol dependent. Of concern is also that similar community descriptions obtained for upper respiratory tract samples across protocols may mistakenly be interpreted as evidence that datasets are comparable also for lower respiratory tract samples. Our findings also lead us to question the conclusions made in studies where similar PCR reagents have been used. Dickson et al. [12] have for example suggested that *Escherichia coli* may be a significant lung pathogen that has previously gone undetected using culture-based techniques. Our results open for interpreting the bacterium as a contaminant introduced with the recombinant DNA polymerase used in the PCR.

We acknowledge that there are limitations to our study. First of all, we did not collect control samples from the bronchoscope working channel used to obtain PBAL and PSB samples. We have however conducted such analyses in our previous publication [3], and shown the DNA extraction kit is the main contamination source for setup 1. For the current work, we focus on PCR protocol steps post-DNA extraction. Because the same DNA extracts were used as input to the three library preparation setups being compared, contamination from earlier steps of the pipeline would affect samples similarly in the different setups. Another limitation is that library preparation and sequencing for each setup was not repeated, and assessement of reliability therefore limited. However, our analyses of the mock community sample, for which was included on all three setups (and processed four times through setup 1), indicated that valid comparisons could be made across setups.

## Conclusion

Our findings show that choice of protocol for library preparation and sequencing (1- or 2- steps of PCR) will have an impact on the analyses of the airway microbiome. Upper airway samples (high biomass) were less impacted than lower airway samples (low biomass), indicating that protocol bias is related to sample biomass. This did not appear to be associated with differences in contamination levels when following a longer or shorter protocol, but rather to mechanisms related to the PCR, for which more research is required. These methodological limitations likely explain the variable conclusions across studies of the airway microbiome (e.g. for comparisons of upper and lower airway samples). Differences in targeted amplicon region (16S rRNA gene V3 V4 versus V4) did not appear have a great impact on final bacterial community descriptions, although greater taxonomic resolution was observed when targeting the longer V3 V4 region.

## Methods

### Study samples

The 23 study subjects were chosen from the Bergen COPD Microbiome Study (short name “MicroCOPD”) for representation of both healthy (*n* = 9) and diseased (asthma (*n* = 4), COPD (*n* = 10)) states. Out of the 350 study subjects included in the MicroCOPD study (with samples dispersed across over 30 sequencing runs), the subset of subjects included in the current investigation were chosen in order to minimize the spread of samples across multiple runs. Details on the MicroCOPD study design and bronchoschopy procedures have been previously published [26]. The MicroCOPD study was approved by the regional ethical committee (REK-Vest, case # 2011–1307), and all subjects signed written informed consent.

In brief, voluntary bronchoscopies were performed on adult subjects (with and without obstructive lung disease) recruited from Western Norway between 2013 and 2015, at the Department of Thoracic Medicine, Haukeland University Hospital. Subjects were examined in the stable state and were not to have received antibiotics at minimum 2 weeks prior to the procedure. Samples collected under each procedure included the first and second fraction of 2 × 50 mL protected (through a sterile inner catheter passed through the scope channel) bronchoalveolar lavage (PBAL1 and PBAL2) from the right middle lobe, three protected specimen brushes sampled from the right lower lobe (PSB), an oral wash (OW) sample, and a negative control sample (NCS) taken from the sterile bottle of phosphate buffered saline directly; the same fluid used for BAL sampling, OW, and dissolution of the PSBs.

We also included a mock community sample, obtained through BEI Resources NIAID, NIH as part of the Human Microbiome Project: Genomic DNA from Microbial Mock Community B (Staggered, Low Concentration), v5.2L, for 16S rRNA Gene Sequencing, HM783D.

### Bacterial DNA extraction

Bacterial DNA extraction was performed first by treatment with lytic enzymes mutanolysin, lysozyme and lysostaphin (all from Sigma-Aldrich, St. Louis, MO, USA) and subsequently by processing through the Fast DNA Spin Kit (MP Biomedcals, LLC, Solon, OH, USA) following the manufacturer’s instructions. The sample volume used as input into the DNA extraction protocol varied with sample type; 450 μl for PSB and NCS and 1800 μl for OW and PBAL.

### Library preparation for MiSeq sequencing

We processed the same DNA extracts through three different library preparation setups for MiSeq sequencing of the bacterial 16S rRNA marker gene: Setup 1 (2-step PCR; 16S rRNA gene region V3 V4); Setup 2 (2-step PCR; 16S rRNA gene region V4); Setup 3 (1-step PCR; 16S rRNA gene region V4). Setups 1 and 2, were based on the 2-step PCR protocol described in the Illumina 16S Metagenomic Sequencing Library Preparation guide (Part no. 15044223 Rev. B). In the first PCR, the 16S rRNA gene regions V3 V4 (setup 1) and V4 (setup 2) were targeted using primers (gene specific sequences are underlined):

Setup 1:

5′-TCGTCGGCAGCGTCAGATGTGTATAAGAGACAGCCTACGGGNGGCWGCAG-3′ and.

5′-GTCTCGTGGGCTCGGAGATGTGTATAAGAGACAGGACTACHVGGGTATCTAATCC-3′.

Setup 2:

5’TCGTCGGCAGCGTCAGATGTGTATAAGAGACAGGTGCCAGCMGCCGCGGTAA3´ and.

5’GTCTCGTGGGCTCGGAGATGTGTATAAGAGACAGGGACTACHVGGGTWTCTAAT3´.

PCR cycling was performed with an initial cycle at 95 °C for 3 min followed by 45 cycles of 95 °C for 30s, 55 °C for 30 s (setup1)/ 50 °C (setup 2), 72 °C for 30 s and a final extension cycle at 72 °C for 5 min. In the second PCR (8 cycles), index sequences were added to the ends of the amplicons generated in the first PCR, using primers from the Nextera XT Index Kit (Illumina Inc., San Diego. CA, USA). Amplifications were performed using the Kappa HiFi HotStart ReadyMix (KAPA Biosystems, USA). Setup 3 was based on the 1-step PCR protocol described in Kozich et al. [24], with modifications (see Additional File 7: Supplementary Methods). The primers used targeted the 16S rRNA gene region V4 and consisted of both gene specific sequences (underlined) and index sequences (N):

Setup 3: 5’AATGATACGGCGACCACCGAGATCTACACNNNNNNNNTATGGTAATTGTGTGCCAGCMGCCGCGGTAA3´.

5’CAAGCAGAAGACGGCATACGAGATNNNNNNNNAGTCAGTCAGCCGGACTACHVGGGTWTCTAAT3´.

PCR cycling was performed with an initial cycle at 95 °C for 2 min followed by 45 cycles of 95 °C for 20 s, 55 °C for 15 s, 72 °C for 5 min and a final extension cycle at 72 °C for 5 min. Amplifications were performed using the recombinant DNA polymerse Accuprime Pfx Super Mix (Thermo Fisher Scientific, USA).

### Bioinformatics

**General Steps.** Sequences were processed using *plugin* tools available within the Quantitative Insights Into Microbial Ecology (QIIME2) bioinformatic package (release 2019.1). Two fastq-files per sample (demulitiplexed, paired-end reads) were imported into the QIIME2 environment. Using the *dada2 denoise-paired* plugin i) primer sequences and low quality bases at read-ends were trimmed off, ii) paired-end reads were joined, iii) chimeras discarded and iv) amplicon sequence variants (ASVs) inferred [25, 28]. Additional chimera filtering was performed using the *vsearch uchime-denovo* plugin [38]. ASVs with fewer sequences than 0.005% of the total number of sequences and ASVs not found in at least two samples were then discarded [27]. Taxonomy was assigned using the *feature-classifier classify-sklearn* plugin together with a Naïve Bayes classifier that had been pre-trained on the full-length Greengenes 13_8 99% OTU reference database (available on qiime2.org). ASVs classified as mitochondria, chloroplasts or archaea were discarded together with classifications that ended above the phylum level. Contaminant ASVs identified using the Decontam package in R were then discarded [32]. The Decontam method “either” (threshold = 0.5) was chosen based on our previous work [3]. As the study samples were found across multiple sequencing runs, bioinformatics processing of samples was performed in batches according to run number. Samples not included in the study, but present on the same run were also included in the pipeline to optimize performance of run specific algorithms (e.g. DADA2 and Decontam). **Analyses.** Analysis on taxonomic composition was performed in Excel on ASV tables generated at various stages of the bioinformatic pipeline. Analyses on procedural samples (PSB, PBAL, OW) were performed on the ASV table processed through all general steps described above. Analyses on the top 20 ASVs found in NCS and in PCR water controls, were based on the ASV table processed through all steps in the pipeline except removal of contaminants identified in Decontam. For analyses on mock community samples, processing steps were limited to DADA2, VSEARCH and removal of ASVs not classified at minimum to phylum level. Analyses of beta-diversity were conducted using PCoA on *unweighted* UniFrac distances. The *unweighted* UniFrac metric scores samples with bacterial communities found at similar positions within the phylogenetic tree, as more similar than samples with bacterial communities found at different positions within the tree. The (dis)similarity between samples is visualized in principal coordinates of analysis (PCoA) space, with samples similar in bacterial composition plotted closer together. The *unweighted* UniFrac metric was chosen to ensure that the less abundant ASVs would have equal impact on the clustering pattern as the high abundant ASVs.

## Supplementary Information

**Additional file 1: Fig. S1.** Comparison of the number of sequences and amplicon sequence variants (ASVs) retained at each bioinformatic filtering step for procedural samples (PSB, PBAL, OW, NCS) collected from 14 participants (*n* = 56).

**Additional file 2: Table S1.** The table presents an overview of the sequence count per ASV obtained after V3 V4 sequencing of mock community sample HM-783D following setup 1.

**Additional file 3: Table S2.** The table presents an overview of the sequence count per ASV obtained after V4 sequencing of mock community sample HM-783D following setup 2.

**Additional file 4: Table S3**. The table presents an overview of the sequence count per ASV obtained after V4 sequencing of mock community sample HM-783D following setup 3.

**Additional file 5: Table S4.** The table presents an overview of the relative abundance of ASVs mapping to *Enterobacteriaceae* per sample across all sample types and setups.

**Additional file 6: Fig. S2.** Principal coordinates analysis on *unweighted* UniFrac distances for procedural samples sequenced following each setup before the removal of Decontam contaminants.

**Additional file 7: Supplementary Methods.** The file provides a detailed description of the mock community HMD 783-D, protocols for sequencing.

**Additional file 8: Fig. S3.** Principal coordinates analysis on *weighted* UniFrac distances for procedural samples sequenced following setup 2 (sphere) and 3 (diamond) *before* the removal of Decontam contaminants.

**Additional file 9: Fig. S4.** Principal coordinates analysis on *weighted* UniFrac distances for procedural samples sequenced following setup 2 (sphere) and 3 (diamond) *after* the removal of Decontam contaminants.

## Data Availability

Fastq files and metadata are available at 10.5061/dryad.8cz8w9gnt. Protocols and laboratory materials used in the MicroCOPD study are available at dx.doi.org/10.17504/protocols.io.2sygefw.

## Abbreviations

ASV: Amplicon sequence variant
COPD: Chronic obstructive pulmonary disease
EMP: Earth microbiome project
HMP: Human microbiome project
NCS: Negative control sample
OTU: Operational taxonomic unit
OW: Oral wash
PBAL: Protected bronchoalveolar lavage
PSB: Protected specimen brushes
QIIME: Quantitative insights into microbial ecology
