# Supplementary material for: Exploring protocol bias in airway microbiome studies: one versus two PCR steps and 16S rRNA gene region V3 V4 versus V4
Source: BMC Genomics. 2021 Jan 4;22:3. doi: 10.1186/s12864-020-07252-z (PMC7784388; doi:10.1186/s12864-020-07252-z)
Supplement: Supplementary file 1 — Additional file 1: Fig. S1. Comparison of the number of sequences and amplicon sequence variants (ASVs) retained at each bioinformatic filtering step for procedural samples (PSB, PBAL, OW, NCS) collected from 14 participants (n = 56). [file 12864_2020_7252_MOESM1_ESM.docx]

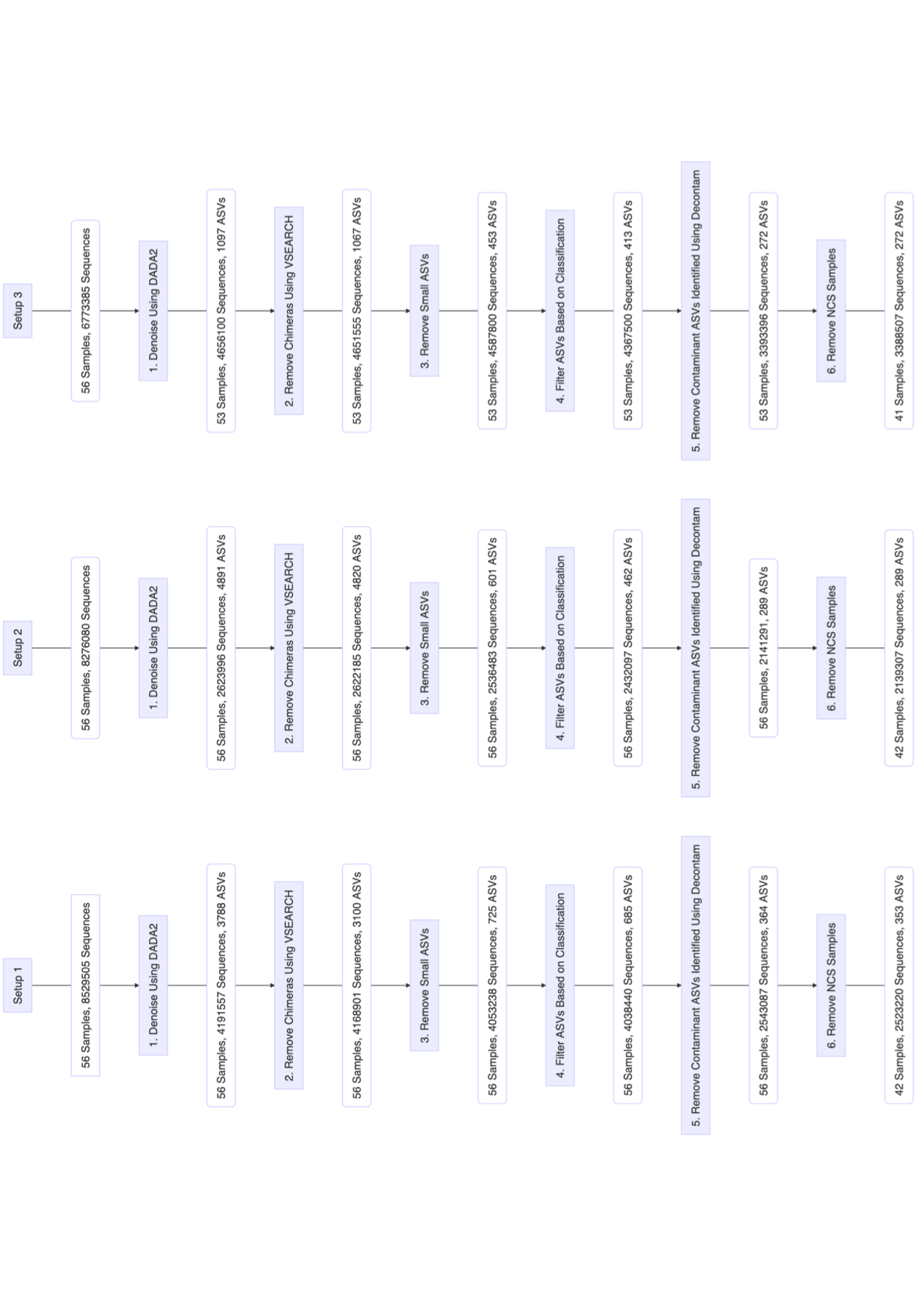
Additional File 1: Figure S.1.

**Figure S.1.** Comparison of the number of sequences and amplicon sequence variants (ASVs), retained at each bioinformatic filtering step for procedural samples (PSB, PBAL, OW, NCS) collected from 14 participants (n=56). Setup 1 (2-step PCR; V3 V4 region), Setup 2 (2-step PCR; V4 region), Setup 3 (1-step PCR; V4 region).
