# Supplementary material for: Exploring protocol bias in airway microbiome studies: one versus two PCR steps and 16S rRNA gene region V3 V4 versus V4
Source: BMC Genomics. 2021 Jan 4;22:3. doi: 10.1186/s12864-020-07252-z (PMC7784388; doi:10.1186/s12864-020-07252-z)
Supplement: Supplementary file 6 — Additional file 6: Fig. S2. Principal coordinates analysis on unweighted UniFrac distances for procedural samples sequenced following each setup before the removal of Decontam contaminants. [file 12864_2020_7252_MOESM6_ESM.docx]

1.
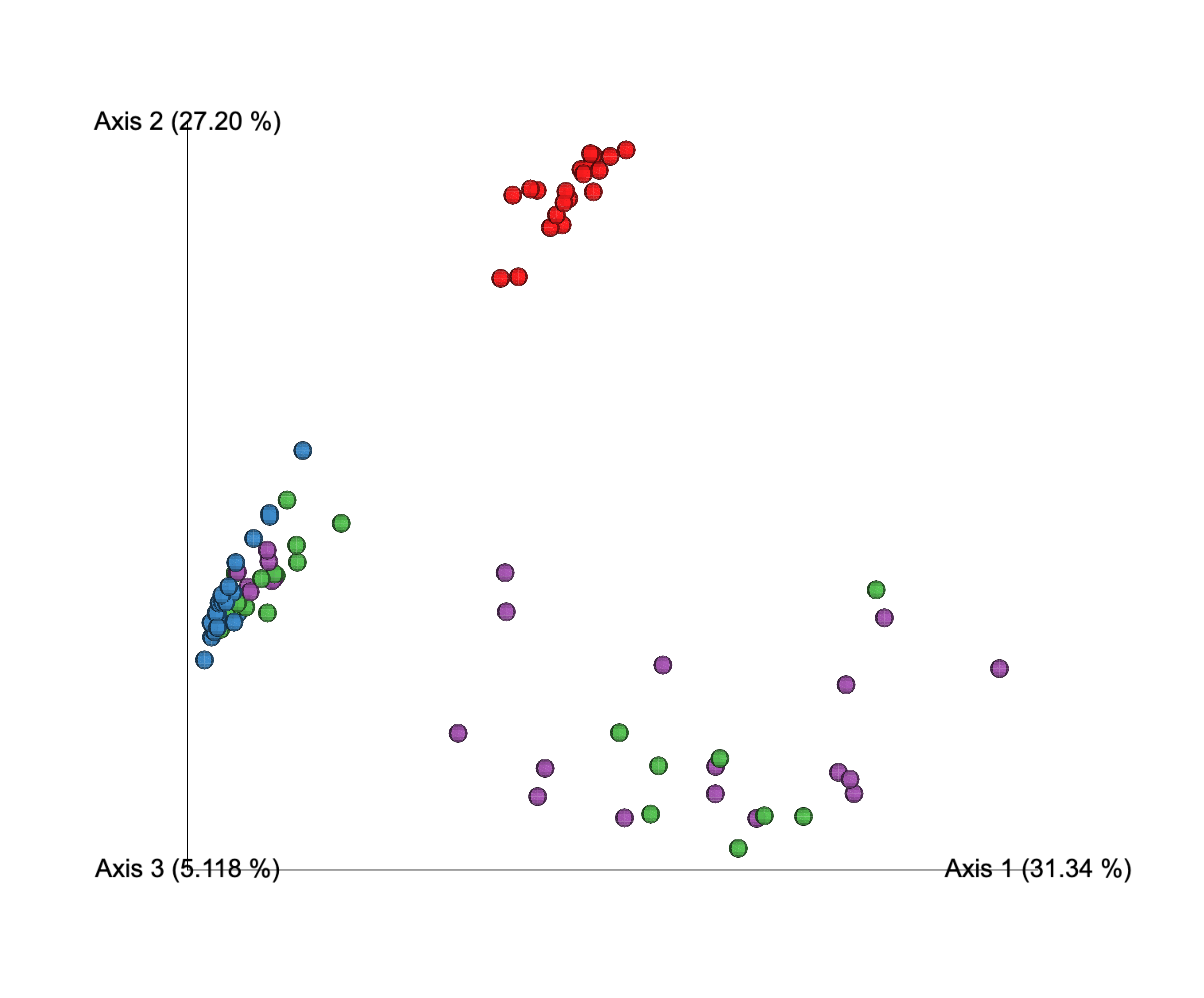
**Setup 1**
2. **Setup 2**


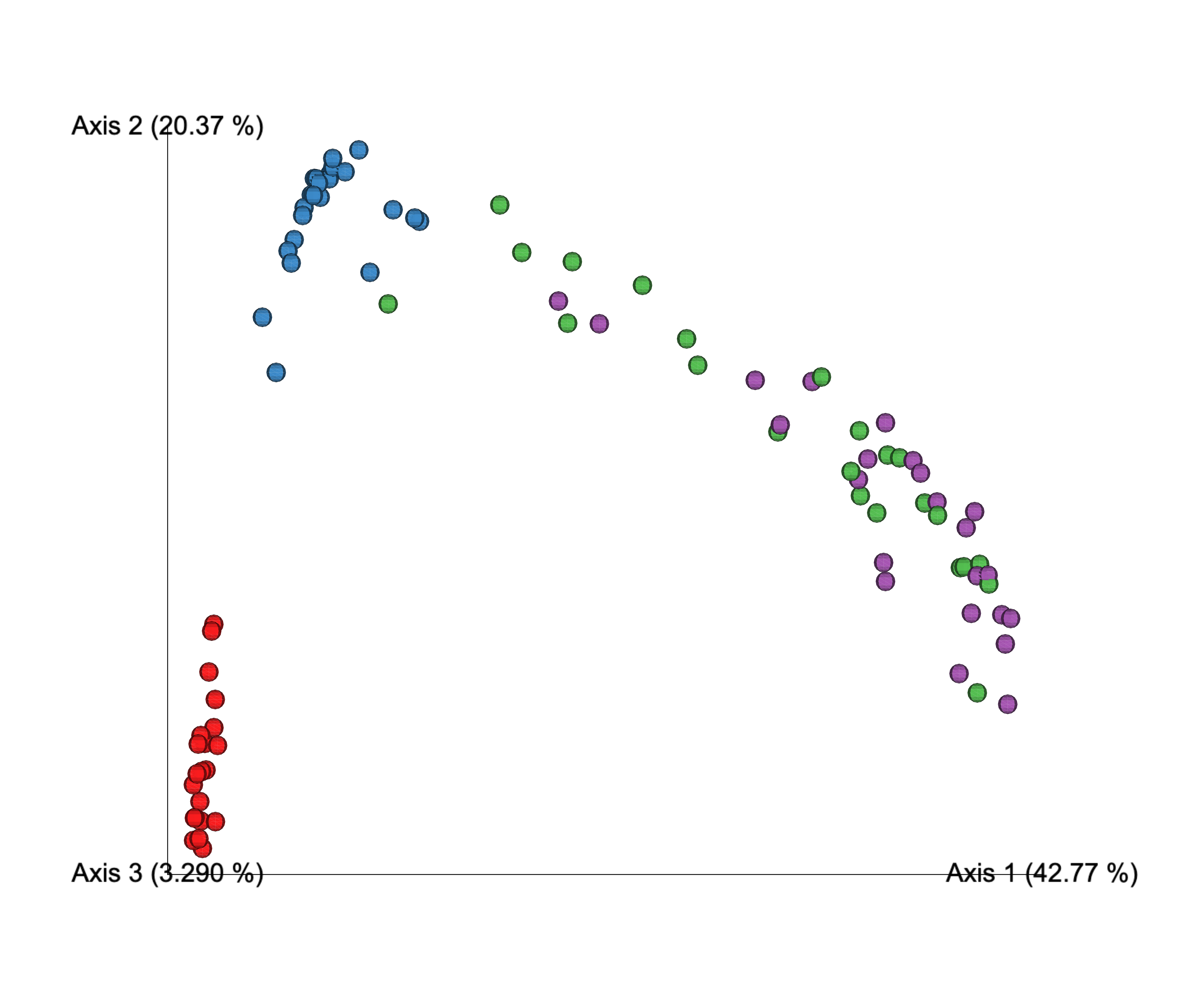


1. **Setup 3**


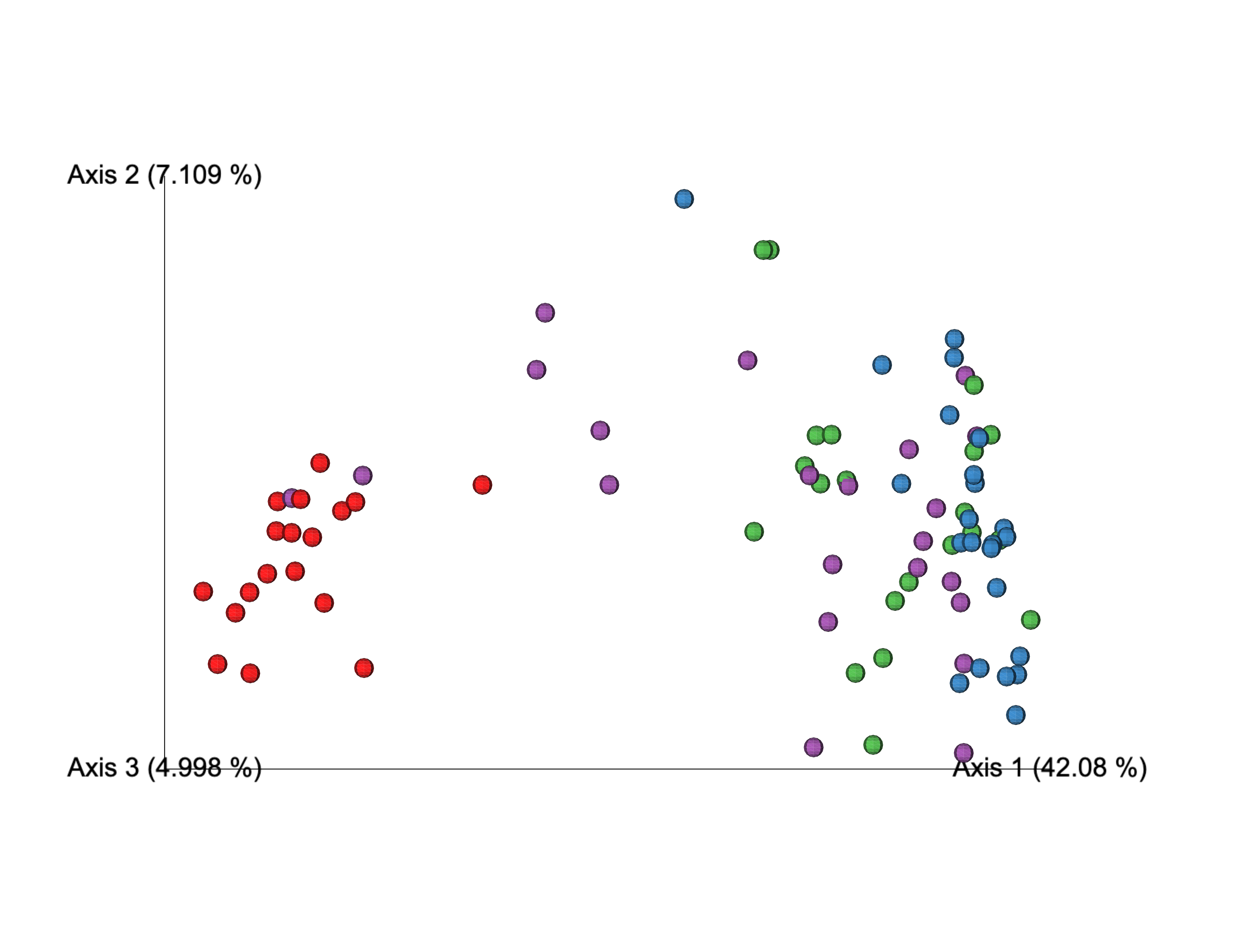


**Supplementary Figure 2**. Principal coordinates analysis on unweighted UniFrac distances for procedural samples sequenced following each setup before the removal of Decontam contaminants. A. Setup 1 (OW: n=22; PBAL: n=23; PSB: n=23; NCS: n=20). B. Setup 2 (OW: n=23; PBAL: n=23; PSB: n= 23; NCS: n=21). C. Setup 3 (OW: n=23; PBAL: n=21; PSB: n=22; NCS: n=18). Setup 1 (2-step PCR; V3 V4 region); Setup 2 (2-step PCR; V4 region); Setup 3 (1-step PCR; V4 region). Rarefaction depth: 1066 sequences. Oral Wash (OW): blue; Protected bronchoalveolar lavage (PBAL): green; Protected specimen brushes (PSB): purple; Negative control samples (NCS): red.
