# Supplementary material for: Exploring protocol bias in airway microbiome studies: one versus two PCR steps and 16S rRNA gene region V3 V4 versus V4
Source: BMC Genomics. 2021 Jan 4;22:3. doi: 10.1186/s12864-020-07252-z (PMC7784388; doi:10.1186/s12864-020-07252-z)
Supplement: Supplementary file 7 — Additional file 7: Supplementary Methods. The file provides a detailed description of the mock community HMD 783-D, protocols for sequencing. [file 12864_2020_7252_MOESM7_ESM.docx]

Additional file 7: Supplementary Methods

Online supplement for

**Exploring protocol bias in airway microbiome studies: One versus two PCR steps and 16S rRNA gene region V3 V4 versus V4**

Christine Drengenes, Tomas ML Eagan, Ingvild Haaland, Harald G Wiker, Rune Nielsen

**Mock Community Sample HM-783D**

The mock community sample was obtained through BEI Resources, NIAID, NIH, as part of the Human Microbiome Project: Genomic DNA from Microbial Mock Community B (Staggered, Low Concentration), v5.2L, for 16S rRNA Gene Sequencing, HM-783D. The input number of 16S rRNA gene operons was given on the certificate of analysis provided by the BEI Resources, and used to calculate the relative abundance of the different bacteria in the sample (Table S.1).

Table S.1. Mock community HM-783D

| **Species** | **Number of operons** | **Relative abundance (%)** |
| --- | --- | --- |
| *Acinetobacter baumannii* | 10000 | 0.22 % |
| *Actinomyces odontolyticus* | 1000 | 0.02 % |
| *Bacillus cereus* | 100000 | 2.19 % |
| *Bacteroides vulgatus* | 1000 | 0.02 % |
| *Clostridium beijerinckii* | 100000 | 2.19 % |
| *Deinococcus radiodurans* | 1000 | 0.02 % |
| *Enterococcus faecalis* | 1000 | 0.02 % |
| *Escherichia coli* | 1000000 | 21.91 % |
| *Helicobacter pylori* | 10000 | 0.22 % |
| *Lactobacillus gasseri* | 10000 | 0.22 % |
| *Listeria monocytogenes* | 10000 | 0.22 % |
| *Neisseria meningitidis* | 10000 | 0.22 % |
| *Propionibacterium acnes* | 10000 | 0.22 % |
| *Pseudomonas aeruginosa* | 100000 | 2.19 % |
| *Rhodobacter sphaeroides* | 1000000 | 21.91 % |
| *Staphylococcus aureus* | 100000 | 2.19 % |
| *Staphylococcus epidermidis* | 1000000 | 21.91 % |
| *Streptococcus agalactiae* | 100000 | 2.19 % |
| *Streptococcus mutans* | 1000000 | 21.91 % |
| *Streptococcus pneumoniae* | 1000 | 0.02 % |

**Library Preparation for MiSeq Sequencing (Setups 1, 2 and 3)**

We compare three different library preparation setups for MiSeq sequencing of the bacterial 16S rRNA gene. The three setups vary with regards to the number of PCR steps (one or two) and the target marker gene region sequenced (16S rRNA gene region V3 V4 or V4): Setup 1 (2-step PCR; region V3 V4); Setup 2 (2-step PCR; region V4); Setup 3 (1-step PCR; region V4).

Setups 1 and 2

Setups 1 and 2 were performed according to the the Illumina 16S Metagenomic Sequencing Library Preparation guide (Part no. 15044223 Rev. B). The protocol consists of two PCR steps; the first for amplification of the target marker gene region to be sequenced and the second for the addition of index sequences required for sample multiplexing.

*Setup 1.* In the first PCR step, the 16S rRNA gene V3 V4 region was targeted using primers:

5′-*TCGTCGGCAGCGTCAGATGTGTATAAGAGACAG*CCTACGGGNGGCWGCAG-3′ (forward) and

5′-*GTCTCGTGGGCTCGGAGATGTGTATAAGAGACAG*GACTACHVGGGTATCTAATCC-3′ (reverse).

Illumina overhang adapter sequences are *italicized*. Gene specific sequences (underlined) are taken from Klindworth *et al.* [1]. Each reaction consisted of 5 µl sample, 12.5 µl KAPA HiFi HotStart ReadyMix (2X) (KAPA Biosystems, USA), 0.5 µl of each primer (10 µM) and 6.5 µl RT-PCR grade water (Thermo Fisher Scientific, USA) for a total volume of 25 µl. PCR cycling was performed using the following program: an initial cycle at 95 °C for 3 minutes, followed by 45 cycles of 95 °C for 30 seconds, 55 °C for 30 seconds, 72 °C for 30 seconds, and a final extension cycle at 72 °C for 5 minutes.

*Setup 2*. In the first PCR step, the 16S rRNA gene V4 region was targeted using primers:

5´-*TCGTCGGCAGCGTCAGATGTGTATAAGAGACAG*GTGCCAGCMGCCGCGGTAA-3´ (forward) and

5´-*GTCTCGTGGGCTCGGAGATGTGTATAAGAGACAG*GGACTACHVGGGTWTCTAAT-3´ (reverse).

Illumina overhang adapter sequences are *italicized*. Gene specific sequences (underlined) are taken from Caporaso *et al.* [2]. Each reaction consisted of 5 µl sample, 12.5 µl KAPA HiFi HotStart ReadyMix (2X), 1.25 µl of each primer (10 µM), and 5 µl RT-PCR grade water (Thermo Fisher Scientific, USA) for a total volume of 25 µl. PCR cycling was performed using the following program: an initial cycle at 95 °C for 3 minutes, followed by 45 cycles of 95 °C for 30 seconds, 50 °C for 30 seconds, 72 °C for 30 seconds, and a final extension cycle at 72 °C for 5 minutes.

For both setups 1 and 2, the second PCR step was performed using primers from the Nextera XT Index kit (Illumina Inc., USA). Each reaction consisted of 5 µl amplicons from PCR step one, 25 µl KAPA HiFi HotStart ReadyMix (2X), 5 µl of each forward and reverse index primer (Nextera XT Kit), and 10 µl RT-PCR grade water (Thermo Fisher Scientific, USA) for a total volume of 50 µl. PCR cycling was performed using the following program: an initial cycle of 95 °C for 3 minutes, followed by 8 cycles of 95 °C for 30 seconds, 55 °C for 30 seconds, 72 °C for 30 seconds, and a final extension cycle at 72 °C for 5 minutes.

Amplicon libraries were quantified using the Qubit dsDNA HS Assay Kit (Life Technologies, USA), normalized to 4 nM and pooled together. The pooled library was denatured with NaOH and diluted to 10 pM. The library was then spiked (15%) with PhiX from the PhiX Control Kit (Illumina). Paired-end sequencing was performed using 2x300 cycles (setup 1)/2x275 cycles (setup 2) on the Illumina MiSeq using reagents from the MiSeq reagent kit v3 (Illumina).

Setup 3

Setup 3 was based on the 1-step PCR protocol described by Kozich *et al*. [3]. The protocol consists of just one PCR step using primers that contain gene targeting sequences, index sequences and illumina sequencing adapter sequences.

The 16S rRNA gene V4 region was targeted using primers 5´-AATGATACGGCGACCACCGAGATCTA CACNNNNNNNNTATGGTAATTGTGTGCCAGCMGCCGCGGTAA-3´ and 5-´CAAGCAGAAGACGGCATACGA GATNNNNNNNNAGTCAGTCAGCCGGACTACHVGGGTWTCTAAT-3´. As detailed in Kozich *et al.* [3], the primers consist of different regions including: the Illumina sequencing adapter sequence, index sequence (NNNNNNNN), pad and linker sequence (reading 5´-3´). The gene specific sequences (underlined) are the same as for the primers used in the sequencing setup 2. Each reaction consisted of 5 µl sample, 18 µl AccuPrime Pfx SuperMix (Thermo Fisher Scientific, USA) and 1 µl of each primer (10 µM) for a total volume of 25 µl. PCR cycling was performed using the following program: an initial cycle at 95 °C for 2 minutes, followed by 45 cycles of 95 °C for 20 seconds, 55 °C for 15 seconds, 72 °C for 5 minutes, and a final extension cycle at 72 °C for 5 minutes. PCR clean-up was performed using Agencourt AMPure XP beads (Beckman Coulter, USA).

Amplicon libraries were quantified using the Qubit dsDNA HS Assay Kit, normalized to 4 nM and pooled together. The pooled library was denatured with NaOH and diluted to 10 pM. The library was spiked (15%) with PhiX from the PhiX Control Kit (Illumina). Paired-end sequencing was performed using 2x250 cycles on the Illumina MiSeq using reagents from the MiSeq reagent kit v3.

**References**

1. Klindworth A, Pruesse E, Schweer T, Peplies J, Quast C, Horn M, et al. Evaluation of general 16S ribosomal RNA gene PCR primers for classical and next-generation sequencing-based diversity studies. Nucleic Acids Res. 2013;41:e1.

2. Caporaso JG, Lauber CL, Walters WA, Berg-Lyons D, Lozupone CA, Turnbaugh PJ, et al. Global patterns of 16S rRNA diversity at a depth of millions of sequences per sample. Proc Natl Acad Sci USA. 2011;108 Suppl 1:4516–22.

3. Kozich JJ, Westcott SL, Baxter NT, Highlander SK, Schloss PD. Development of a Dual-Index Sequencing Strategy and Curation Pipeline for Analyzing Amplicon Sequence Data on the MiSeq Illumina Sequencing Platform. Appl Environ Microbiol. 2013;79:5112–20.
