# Supplementary material for: Exploring protocol bias in airway microbiome studies: one versus two PCR steps and 16S rRNA gene region V3 V4 versus V4
Source: BMC Genomics. 2021 Jan 4;22:3. doi: 10.1186/s12864-020-07252-z (PMC7784388; doi:10.1186/s12864-020-07252-z)
Supplement: Supplementary file 9 — Additional file 9: Fig. S4. Principal coordinates analysis on weighted UniFrac distances for procedural samples sequenced following setup 2 (sphere) and 3 (diamond) after the removal of Decontam contaminants. [file 12864_2020_7252_MOESM9_ESM.docx]

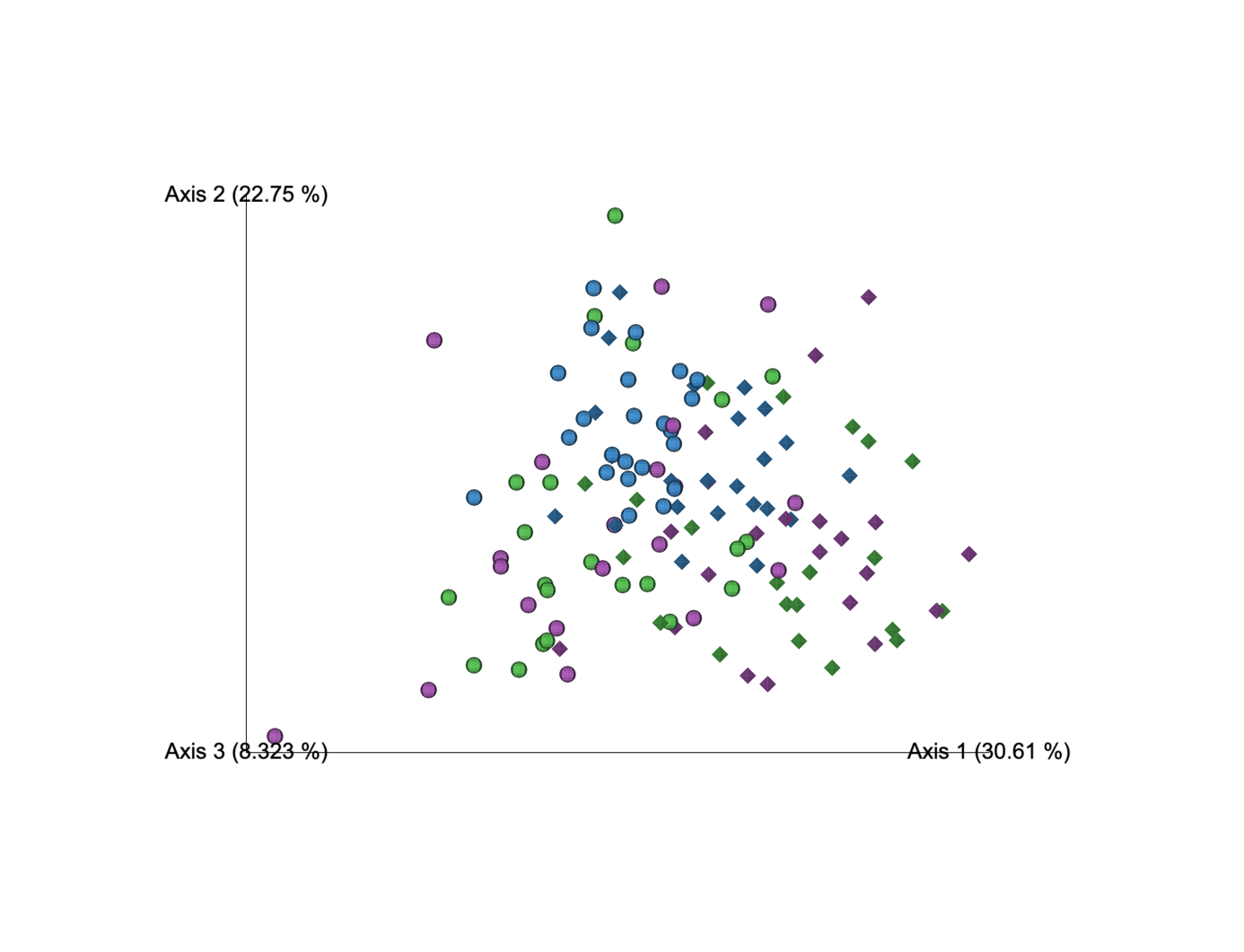


**Supplementary Figure 4.** Principal coordinates anaysis on weighted UniFrac distances for procedural samples sequenced following setup 2 (sphere) and 3 (diamond) *after* the removal of Decontam contaminants. Rarefaction depth: 1139 sequences. Setup 2 samples include OW: n=23; PBAL: n=22; PSB: n=20 and setup 3 samples include OW: n=23; PBAL: n=21; PSB: n=21. Oral wash (OW): blue; Protected bronchoalveolar lavage (PBAL): green; Protected specimen brushes (PSB): purple. Setup 2 (2-step PCR; V4 region), Setup 3 (1-step PCR; V4 region).
